# Protocadherin 17 functions as a tumor suppressor suppressing Wnt/β-catenin signaling and cell metastasis and is frequently methylated in breast cancer

**DOI:** 10.18632/oncotarget.10102

**Published:** 2016-06-16

**Authors:** Xuedong Yin, Tingxiu Xiang, Junhao Mu, Haitao Mao, Lili Li, Xin Huang, Chunhong Li, Yixiao Feng, Xinrong Luo, Yuxian Wei, Weiyan Peng, Guosheng Ren, Qian Tao

**Affiliations:** ^1^ Chongqing Key Laboratory of Molecular Oncology and Epigenetics, The First Affiliated Hospital of Chongqing Medical University, Chongqing, China; ^2^ Cancer Epigenetics Laboratory, Department of Clinical Oncology, State Key Laboratory of Oncology in South China, Sir YK Pao Center for Cancer and Li Ka Shing Institute of Health Sciences, The Chinese University of Hong Kong and CUHK Shenzhen Research Institute, Hong Kong; ^3^ Oncology Department, Suining Sichuan Center Hospital, Sichuan, China

**Keywords:** tumor suppressor, PCDH17, methylation, cancer, β-catenin

## Abstract

Protocadherins play important roles in the regulation of cell adhesion and signaling transduction. Aberrant expression of protocadherins has been shown to be associated with multiple tumorigenesis. We previously identified *PCDH17*, encoding protocadherin 17, as a frequently methylated and downregulated tumor suppressor gene (TSG) in gastric and colorectal cancers. Here, we examined the abnormalities and functions of *PCDH17* in breast cancer pathogenesis. We used PCR and immunohistochemistry to check its expression pattern in breast tumor cell lines and primary tumors. Methylation-specific PCR (MSP) was applied to examine its promoter methylation status in breast tumor cell lines and primary tumors. The biological functions of *PCDH17* in breast tumor cells were assessed using *in vitro* and *in vivo* assays. We found that *PCDH17* was frequently downregulated or silenced in 78% (7/9) of breast tumor cell lines, as well as 89% (32/36) of primary tumors. Downregulation of *PCDH17* in breast cancer was mainly due to the methylation of its promoter. Ectopic expression of *PCDH17* in breast tumor cells inhibited cell proliferation and mobility through arresting cell cycle and inducing apoptosis. In breast tumor cells, *PCDH17* significantly suppressed the active β-catenin level and its downstream target gene expression. Thus, we found that *PCDH17* functions as a tumor suppressor inhibiting Wnt/β-catenin signaling and metastasis in breast cancer but is frequently methylated in primary tumors which could be a potential biomarker.

## INTRODUCTION

Breast cancer is the major cause of cancer-related morbidity and mortality in females worldwide [1]. Although many drugs and therapeutic regimens have been developed for the treatment of breast cancer, there is no cure for patients with systemic metastatic breast cancer. Diagnosis of breast cancer during its early stages correlates with increased overall survival rate. Therefore, females with high risk of developing breast cancer are encouraged to undergo mammography on an annual basis. However, tumors could evade detection by conventional physical examinations during their early stages.

Promoter methylation of tumor suppressor genes (TSGs) has been widely investigated and found to promote breast tumorigenesis. The expression of many TSGs, such as *BRCA1*, *ERa* and *RASSF1A* are downregulated in breast cancer, by promoter methylation [2–5]. Our group also identified some TSGs methylation in breast cancer, including *DACT1*, *ZNF545*, *PLCD1*, *UCHL1*, *DKK3*, which contributes to tumor malignant progression [6–10]. Promoter methylation is the primary mechanism for transcriptional repression of TSGs, thus could serve as a biomarker for early detection of breast cancer [2, 11–14].

Expression profiling and epigenetic studies have been conducted to identify candidate TSGs. The *PCDH17* gene, encoding the protocadherin 17 (PCDH17) protein, has been identified as a TSG [15]. *PCDH17*, located on chromosome 13q21.1, is frequently downregulated. Hypermethylation of its promoter has been found in esophageal, renal, and digestive carcinomas [15–17]. It is unclear whether *PCDH17* acts as a TSG in breast cancer. Therefore, we investigated *PCDH17* expression levels and the methylation status of its promoter in breast tumor cell lines and primary tissues, as well as its biological functions in breast tumorigenesis.

## RESULTS

### PCDH17 expression is downregulated in both breast tumor cell lines and primary breast tumors

**Figure 1:**
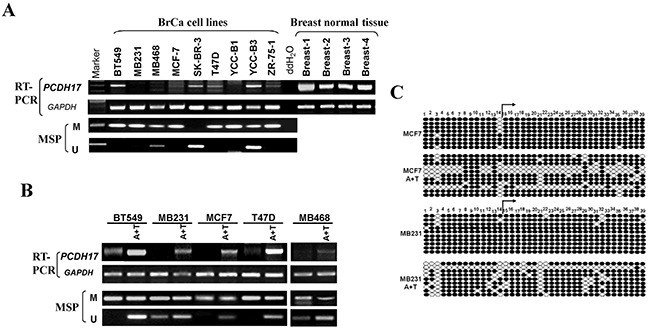
Expression and methylation of PCDH17 in breast tumor cells **A.** Expression of *PCDH17* in breast tumor cell lines and human normal breast tissues detected by semi-quantitative RT-PCR, with GAPDH as a control. Promoter methylation of *PCDH17* was detected by MSP in breast tumor cells. **B.** The effects of pharmacologic demethylation with 5-aza-2′-deoxycytidine (Aza) and trichostatin A (TSA) treatment in BT549, MB231, MCF7, T47D, and MB468 breast tumor cells were examined. M: methylated; U: unmethylated. **C.** Bisulfite sequencing of MCF7 and MB231 cell line before and after A+T treatment. Filled circle, methylated; open circle, unmethylated.

**Figure 2:**
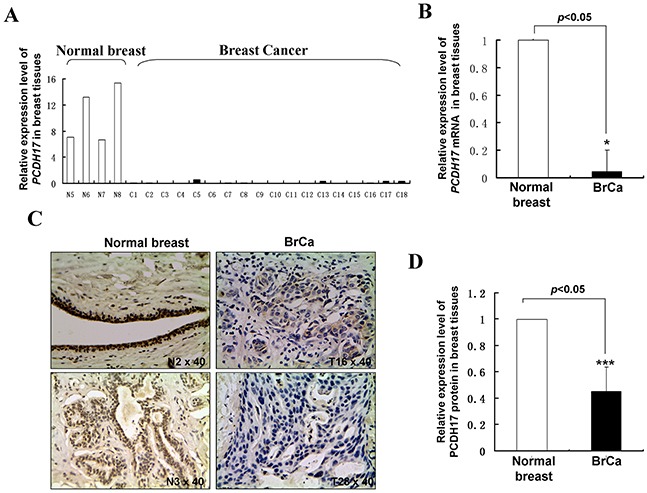
*PCDH17* expression in breast tumor tissues **A, B.** mRNA expression levels of *PCDH17* in human normal breast tissues and breast tumor tissues were detected by qPCR (*p<0.05). BrCa: breast cancer tissues. **C.** Representative immunohistochemistry (IHC) staining for PCDH17 in human normal breast tissues and breast tumor tissues. **D.** Quantitative analysis of mean optical density (MOD) of PCDH17 expression in two groups was shown as values of mean±SD (***p<0.001).

We examined expression levels of *PCDH17* in breast tumor cell lines, normal breast tissue samples, and breast tumor samples using semi-quantitative RT-PCR, quantitative real-time PCR (qPCR) and immunohistochemistry analysis. *PCDH17* expression was markedly repressed in seven of the nine breast tumor cell lines tested, and weakly expressed in BT549, YCC-B3 and Sk-BR-3 cell lines. In contrast, strong expression of *PCDH17* was found in normal breast tissues (Figure 1A). The average mRNA expression level of *PCDH17* in 18 breast tumor samples was significantly decreased compared with normal tissues (*p* < 0.05, Figure 2A, 2B). Immunohistochemistry results showed that protein expression levels of PCDH17 in breast tumor tissues were repressed in 89% (32/36) of cases, in comparison with those seen in normal tissues (*p* < 0.01, Figure 2C, 2D). We failed to observe any correlation between PCDH17 expression levels and clinicopathological characteristics of breast cancer. These findings suggest that *PCDH17* expression is downregulated in breast tumor cell lines and primary breast tumors.

### Promoter methylation contributes to PCDH17 downregulation in breast tumor cell lines

We next determined whether promoter methylation is involved in *PCDH17* downregulation in breast cancer. Typical CpG islands were found in the promoter regions and exon 1 of *PCDH17* using CpG island analysis software (http://cpgislands.usc.edu/) (data not shown). We used methylation-specific PCR (MSP) to analyze *PCDH17* promoter methylation status in nine breast tumor cell lines. Methylation of the *PCDH17* promoter was observed in seven of the nine breast tumor cell lines, which was consistent with its low expression or absence (Figure 1A). To clarify whether *PCDH17* silencing was a direct result of promoter methylation, we treated BT549, MB231, MCF7, T47D, and MB468 cell lines with Aza and TSA, and then performed MSP analysis. We observed that *PCDH17* expression was rescued following the treatment with Aza and TSA, along with demethylation of the *PCDH17* promoter, as demonstrated by MSP and bisulfite sequencing (Figure 1B, 1C). These results suggest that promoter methylation is directly responsible for *PCDH17* downregulation in breast tumor cells.

**Figure 3:**
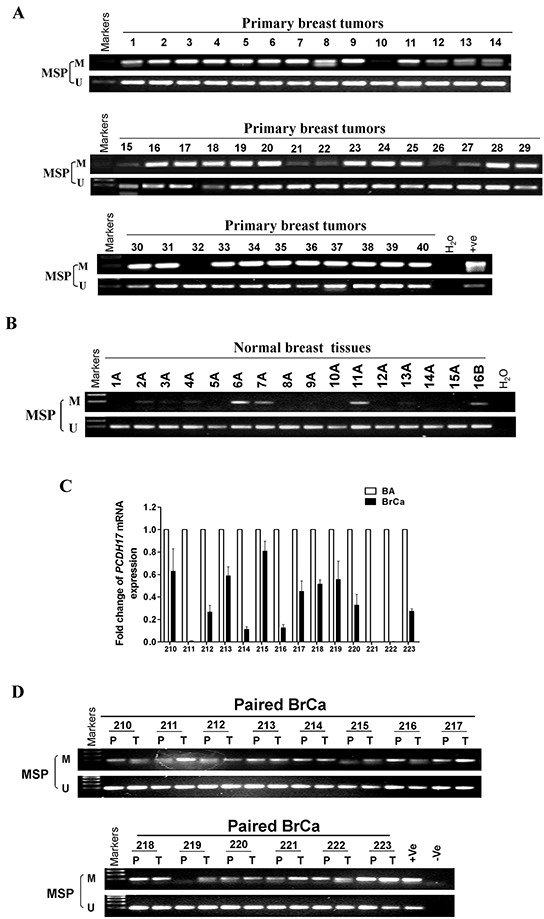
*PCDH17* methylation in breast tumor tissues Methylation of the *PCDH17* promoter in **A.** breast tumor tissues **B.** normal breast tissues was measured by MSP. M: methylated; U: unmethylated. **C.** *PCDH17* expression levels in paired breast tumor and surgical margin tissues were detected by qPCR. BA: breast tumor adjacent tissues; BrCa: breast cancer tissues. **D.** Promoter methylation of paired breast tumor and surgical margin tissues were detected by MSP. M: methylated; U: unmethylated.

We also investigated *PCDH17* promoter methylation in breast tumor tissues. We observed that *PCDH17* was methylated in 93.3% (97/104) of primary breast tumor tissues, and 25% (4/16) of normal breast tissues (Table 1, Figure 3A, 3B). 14 pairs of primary breast tumor tissues and surgical margin tissues were also tested for *PCDH17* expression and promoter methylation by qPCR and MSP respectively. All 14 tumors have lower *PCDH17* mRNA expression compared with their paired surgical margin tissues (Figure 3C), while displaying a higher level of promoter methylation (Figure 3D). However, there was no correlation between *PCDH17* promoter methylation and clinicopathological characteristics (data not shown).

**Table 1:** Promoter methylation status of *PCDH17* in primary breast tumors

Tissue	Samples (number)	*PCDH17* promoter		Frequency of methylation	*p* value
		Methylated	Unmethylated		
Breast tumor	104	97	7	93.2%	3.31E-12
Normal breast	16	4	12	25%	

### PCDH17 inhibits cell proliferation in breast tumor cells

**Figure 4:**
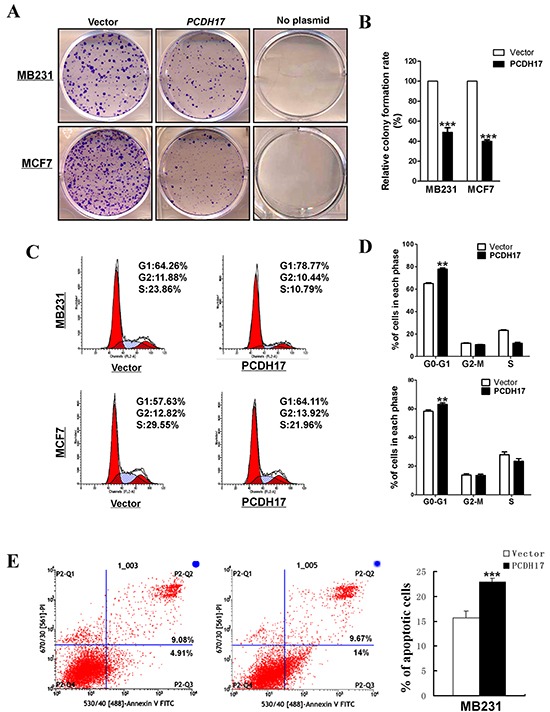
The inhibitory effect of *PCDH17* on proliferation in breast tumor cells **A.** Representative colony formation assay in vector- and *PCDH17*-transfected MB231 and MCF7 cells. **B.** Quantitative analysis of colony formation. The values were shown as the mean ± SD from three independent experiments (***p< 0.001). **C.** Cell cycle distribution of vector- and *PCDH17*-transfected MB231 and MCF7 cells was detected by flow cytometric analysis. **D.** Quantitative analysis of flow cytometric assays (**p<0.01). **E.** Apoptosis of vector- and *PCDH17*-transfected MB231 cells was detected by Annexin V-FITC/PI staining (***p< 0.001).

*PCDH17* silencing *via* promoter methylation in breast cancer suggests that it might function as a TSG. To investigate the effects of *PCDH17* on cell proliferation, we conducted colony formation assays. Ectopic expression of *PCDH17* suppressed the colony formation abilities of breast tumor cells (MB231 and MCF7), with an approximate 50–60% reduction in colony numbers in comparison with cells transfected with control vectors (Figure 4A, 4B). To explore whether *PCDH17* affected cell cycle and apoptosis of breast tumor cells, flow cytometry analyses were performed. Cell cycle results showed that *PCDH17*-transfected cells were arrested more often in the G0/G1 phase than those transfected with the empty control vector (*p* < 0.01, Figure 4C, 4D), accompanied by a decrease in the number of *PCDH17*-expressing cells in the S and G2/M phases (Figure 4C, 4D). Western blot further confirmed increased levels of p21 and p27 protein expression, and decreased levels of cyclin D1 and cyclin B1 protein expression in *PCDH17*-transfected cells (Figure 6B). Annexin V-FITC/PI staining results indicated that *PCDH17* has a pro-apoptotic effect on breast tumor cells (*p* < 0.001, Figure 4E).

### PCDH17 suppresses epithelial-mesenchymal transition (EMT) and migration of breast tumor cells

**Figure 5:**
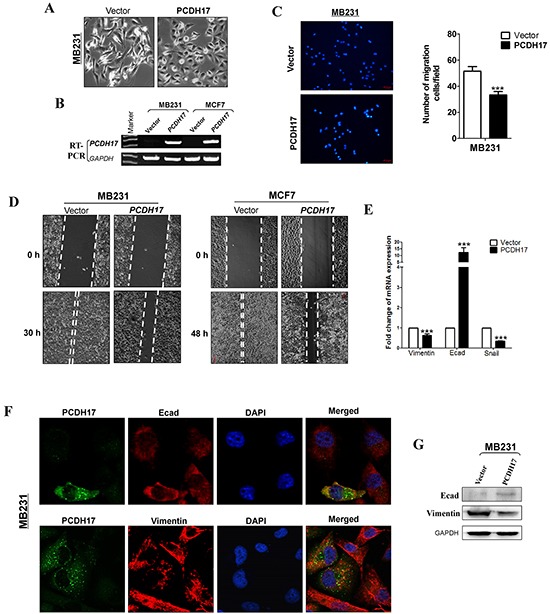
Ectopic expression of *PCDH17* inhibited migration of MB231 and MCF7 cells **A.** Morphology changes of MB231 cells transfected with empty vectors or *PCDH17* by phase-contrast microscopy (Magnification, ×400). **B.** Ectopic expression of *PCDH17* in MB231 and MCF7 cells was detected by RT-PCR. **C.** Cell motility of vector- and *PCDH17*-transfected cells (MB231 and MCF7) were tested by transwell. Results from three independent experiments were quantified as mean ± SD. **D.** Cell motility of vector- and *PCDH17*-transfected cells (MB231 and MCF7) were tested by wound healing assay. Representative images were shown from three independent experiments. **E.** mRNA expression levels of Vimentin, E-cadherin, and Snail were examined by qPCR in MB231 cells (***p< 0.001). **F.** Double immunofluorescence staining of PCDH17 with E-cadherin (Ecad) and Vimentin in MB231 cells. **G.** Protein expression levels of Ecad and Vimentin in vector- and *PCDH17*-transfected MB231 cells.

We found that ectopic *PCDH17* expression caused significant changes in cell morphology. Vector-transfected cells showed spindle-shaped morphology like fibroblasts, while a large proportion of *PCDH17*-transfected cells are elliptical shaped, suggesting that *PCDH17* most likely reversed tumor cell EMT (Figure 5A). qPCR showed that expression of the epithelial marker E-cadherin was upregulated in *PCDH17*-expressing cells, while Vimentin and SNAIL were repressed, providing more evidence that *PCDH17* represses EMT (Figure 5E). Western blot confirmed the changes of E-cadherin and Vimentin on the protein level (Figure 5G). Furthermore, immunofluorescence staining (IF) showed a significant co-expression of protocadherin 17 with E-cadherin, while Vimentin expression was depleted in *PCDH17*-transfected cells (Figure 5F). Transwell assay showed that the number of migrated cells was significantly reduced in cells with ectopic expression of PCDH17 than the control cells (*p*<0.01, Figure 5B, 5C). Furthermore, wound healing assay, at 30 h and 48 h, showed that *PCDH17*- expressing cells migrated along the edges of wounds at a much slower rate than those transfected with empty control vectors, indicating that *PCDH17* inhibits cell migration (Figure 5D).

### PCDH17 regulates Wnt/β-catenin signaling in breast tumor cells

**Figure 6:**
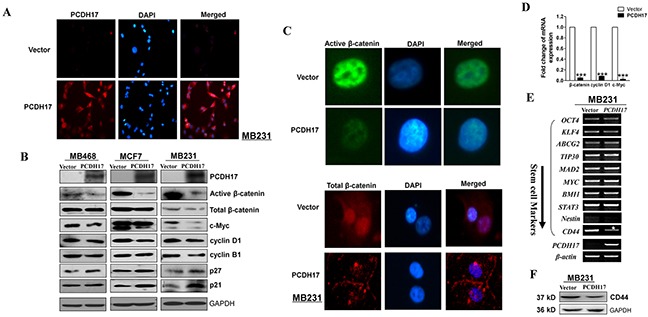
Ectopic expression of *PCDH17* in MB231 and MCF7 cells disrupted Wnt/ β-catenin signaling **A.** Subcellular localization of PCDH17 in MB231 cells by immunofluorescence staining. **B.** Western blot analysis of ß-catenin and its downstream targets in MB468, MCF7, and MB231 cells. **C.** Subcellular localization of active β-catenin and total β-catenin in MB231 cells by immunofluorescence staining. **D.** mRNA expression of β-catenin, cyclin D1, and c-Myc in vector- and *PCDH17*-transfected MB231 cells (***p< 0.001). **E.** Stem cell markers were detected by RT-PCR in vector- and *PCDH17*-transfected MB231 cells. **F.** Protein expression of CD44 in vector- and *PCDH17*-transfected MB231 cells detected by western blot.

The Wnt/β-catenin signaling pathway contributes to breast cancer proliferation and metastasis. Certain protocadherins are capable of directly binding to β-catenin and/or decreasing its activity. Therefore, it is possible that *PCDH17* could be an antagonist of Wnt/β-catenin signaling in breast cancer. We found that protein expression of active β-catenin, but not total β-catenin, was reduced in *PCDH17*-transfected MB231 cells by immunofluorescence (Figure 6A, 6C). Reduced expression of active β-catenin and its downstream target genes c-Myc and cyclin D1 was also observed in *PCDH17*-transfected tumor cells (MB468, MCF7 and MB231) by western blot (Figure 6B). This result was verified by qPCR detection of β-catenin, cyclin D1, and c-Myc mRNA expression in MB231 cells (Figure 6D). We also detected the expression of stemness markers using qPCR. Only *CD44* expression was significantly inhibited by *PCDH17* (Figure 6E). Western blot detection of CD44 corroborated with qPCR (Figure 6F).

### PCDH17 suppresses proliferation of breast tumor cells in vivo

**Figure 7:**
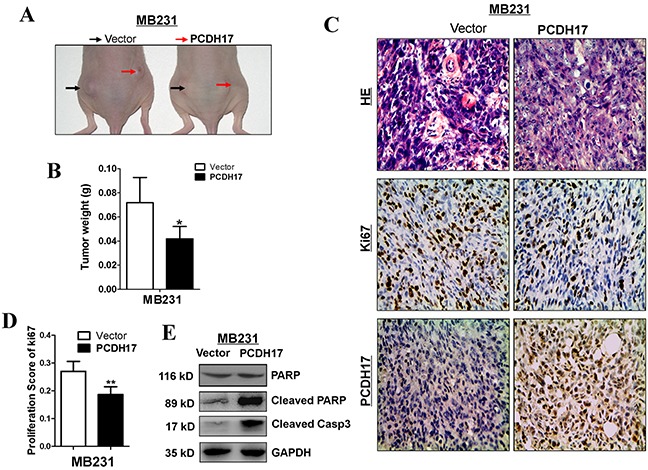
PCDH17 inhibited breast cancer tumorigenicity *in vivo* **A.** Representative image of tumor growth in nude mice. **B.** Tumor weight of *PCDH17*-expressing cells in nude mice compared with vector tumors (6 xenografts per group) (*p<0.05). **C.** Representative images of HE staining and IHC analyses of the expression of PCDH17 and Ki67 in tumors from nude mice (Magnification, × 400). **D.** Proliferation score of tumor xenografts calculated from Ki67 slides. **E.** Detection of apoptosis markers cleaved PARP and cleaved caspase 3 in vector- and *PCDH17*-transfected xenografts.

MB231 cells transfected with empty control vectors or *PCDH17* were injected subcutaneously into nude mice to generate xenograft tumor models. These models were used to evaluate the tumor suppressive function of *PCDH17 in vivo*. The size and weight of tumors induced by *PCDH17*-expressing cells were significantly decreased compared with controls (Figure 7A, 7B). The effect of *PCDH17* on tumor cell growth was also assessed by immunohistochemical staining. Expression level of Ki67 was reduced in tumors where *PCDH17* was present (Figure 7C, 7D). Proliferation of the *PCDH17*-transfected xenografts was suppressed due to escalated apoptosis, as the levels of cleaved poly ADP-ribose polymerase (PARP) and cleaved caspase-3 are significantly higher than the control group (Figure 7E).

## DISCUSSION

Protocadherins are a subfamily of the cadherin superfamily of proteins. However, little is known about their roles in cancers. Protocadherins are predominantly expressed during development of the nervous system, and their functions could be related to the differentiation of embryonic cells [18–20]. Repression of protocadherin expression might contribute to tumorigenesis. It was previously shown that expression levels of protocadherins, such as *PCDH8*, *PCDH10*, and *PCDH20*, were downregulated by promoter methylation in various carcinomas [21–23], which were strongly associated with advanced cancer or poor outcome. Consistent with our findings, other members of the protocadherin family also obstruct tumor progression by antagonizing Wnt/β-catenin pathway in malignancies such as hepatocellular carcinoma and endometrial carcinoma [24, 25]. These studies suggest that protocadherins play crucial roles in the development of cancer. Hence, there is immense clinical value in investigating the expression and promoter methylation of protocadherin genes in breast cancer.

We observed that *PCDH17* were reduced in breast tumor cell lines and primary tumor samples. We found that *PCDH17* expression was downregulated or silenced in all of the cell lines examined. MSP and bisulfite sequencing revealed that *PCDH17* promoter methylation was partially responsible for its downregulation in breast tumor cells. The correlation between promoter methylation and downregulated expression was observed in most, but not all, of the cell lines. The methylation of *PCDH17* promoter regions in BT549 and YCC-B3 cells showed a limited inhibiting effect on mRNA expression. In SK-BR-3 cell lines, *PCDH17* expression was weak, yet, no methylation was observed in SK-BR-3 cell. These might be caused by the heterogeneity of tumor cells, and also suggest that other mechanisms such as histone deacetylation might be involved. Expression of *PCDH17* was repressed in 89% (32/36) of breast tumor tissue samples, indicating that a loss of *PCDH17* expression could lead to breast tumorigenesis. In addition, the *PCDH17* promoter was frequently methylated in breast tumors. Thus, *PCDH17* promoter methylation could be a potential biomarker for the diagnosis of breast cancer.

The biological functions of PCDH17 in breast cancer are unclear. The PCDH17 protein contains six cadherin repeats, an RGD motif in the extracellular domain, and highly conserved motifs (CM1, CM2) in the cytoplasmic domain [26]. Protocadherin 17 was detected during development of the nervous system in humans, and its dysfunction was linked to schizophrenia [26]. Little is known about the molecular mechanisms of PCDH17 during the development of breast cancer. During our investigation, we observed a significant change in the morphology of mesenchymal MB231 cells. The cells displayed epithelial traits after restoring PCDH17 expression. Thus, protocadherin 17 may be able to reverse EMT. IF and western blot results support this hypothesis as epithelial marker E-cadherin was upregulated and mesenchymal marker Vimentin was inhibited by *PCDH17*. *PCDH20* was also able to suppress EMT by enhancing E-cadherin in nasopharyngeal carcinoma [27], which suggests that the protocadherin family plays a regulatory role in the EMT process.

*PCDH17* has been shown to have suppressive effects on the proliferation of tumor cells by inducing apoptosis and cell cycle arrest. We confirmed that ectopic expression of PCDH17 resulted in the arrest of cell cycle, and the induction of cell apoptosis in breast tumor cells. This occurred partially through the upregulated expression of p21 and p27, and reduced expression of cyclin D1 and cyclin B1. *PCDH17* also suppressed β-catenin activity in breast tumor cells, although the molecular mechanism remains unknown and requires further investigation. In a recent study, Wnt/β-catenin signaling was inhibited by *PCDH20* through phosphorylation of GSK-3β in hepatocellular carcinoma [24]. It is possible that they share the same mechanism of suppressing tumor growth.

*PCDH17* also inhibited the expression of c-Myc, another downstream target of β-catenin. This signaling pathway most likely also contributes to the tumor-suppressive effect of PCDH17, as c-Myc is an important oncogenic transcriptional factor [28]. We found that suppression of CD44 is involved in this process. CD44 is a receptor for hyaluronic acid and influences many characteristics of cancer cells, including stemness [29]. This implies that PCDH17 could possibly act as a suppressor of breast cancer stemness and plays a role in the initiation of the disease, which is worth looking into.

In summary, we found that *PCDH17* expression is frequently repressed, or silenced, in breast tumor cell lines when its promoter is methylated. Expression of *PCDH17* could be reactivated by pharmacological demethylation. Downregulation of *PCDH17* and methylation of its promoter were common events in breast cancer. We further confirmed the suppression of *PCDH17* on breast tumor cell proliferation and metastasis. Taken together, these results indicate that *PCDH17* is a functional TSG in breast tumor pathogenesis.

## MATERIALS AND METHODS

### Cell lines and tumor samples

Breast cancer cell lines (MB231, MB468, MCF7, BT549, T47D, SK-BR-3, ZR-75-1, YCC-B1, YCC-B3) were used. Cell lines were maintained in RPMI 1640 (Gibco BRL, Karlsruhe, Germany) supplemented with 10% fetal bovine serum (FBS; Invitrogen, Carlsbad, CA, USA), 100 U/mL penicillin, and 100 U/mL streptomycin at 37°C/5% CO_2_ [8, 30]. Samples of DNA and RNA were obtained from primary breast tumor tissues, paired margin tissues, and normal breast tissues. Fresh cancer tissues and adjacent normal tissues were obtained from patients who underwent primary surgery at the Surgery Department of the First Affiliated Hospital of Chongqing Medical University. Our study was approved by the Institutional Ethics Committees of the First Affiliated Hospital of Chongqing Medical University (Approval Notice 2010/2012(23)). Our experiments were undertaken with the understanding and written consent of each subject.

### DNA and RNA extraction

Genomic DNA was extracted from cell lines and tissues using DNAzol Reagent (Invitrogen, Rockville, MD, USA) and the QIAamp DNA Mini Kit (Qiagen, Hilden, Germany) according to the manufacturers' protocols. Total RNA was isolated from cell lines and tissues using TRI Reagent (Molecular Research Center, Cincinnati, OH, USA). The concentration of RNA samples was determined by spectrophotometry using a NanoDrop 2000 (Thermo Scientific, Wilmington, DE, USA). DNA and RNA integrity were determined by gel electrophoresis. Samples were stored at −80°C until required.

### Immunohistochemical staining

Clinical tissue samples were fixed in 4% paraformaldehyde after surgical procedures. The samples were embedded in paraffin before cutting into 4 μm sections. The sections were dewaxed and rinsed in absolute xylene. The sections were then rehydrated through graded alcohol. Antigen retrieval was performed. To block endogenous peroxidase activity, the sections were incubated in 3% hydrogen peroxide and then washed with phosphate-buffered saline (PBS). Blocking was performed with 5% FBS-PBS solution for 15 min at room temperature. The sections were incubated at 4°C overnight with rabbit monoclonal antibody (PCDH17, 1: 100 dilution; Ki67 Ready-to-use). On the following day, the slides were washed with PBS and incubated with secondary antibody for 15 min at room temperature, followed by color development with Diaminobenzidine (DAB). The cell nuclei were counterstained with hematoxylin. Images were captured with a microscope.

### DNA demethylation

Breast cancer cell lines were subjected to DNA demethylation treatments, as previously described [8]. Cell lines were treated with 10 mM 5-aza-2′-deoxycytidine (Aza) (Sigma-Aldrich, Steinheim, Germany) for 3 days, and then treated with 100 nM trichostatin A (TSA) (Sigma-Aldrich, Steinheim, Germany) for an additional 24 h.

### Semi-quantitative PCR and quantitative PCR (qPCR) assays

For semi-quantitative PCR, the PCDH17 gene was amplified using Go-Taq (Promega) as previously described with GAPDH as a control [31]. Primers used were PCDH17-F: 5′-TGGAGGAGAGGAACGCCATG-3′ and PCDH17-R: 5′-AACAAACTGCTGCCTGCTGC-3′. Quantitative real-time PCR was carried out in the HT7500 system (Applied Biosystems, Foster City, CA, USA) using the SYBR® Green PCR Master Mix (Thermo Fisher Scientific, Hong Kong). The *GAPDH* gene, encoding glyceraldehyde 3-phosphate dehydrogenase, was selected as a reference gene for qPCR assays. Primers used in this study are listed in Table 2.

**Table 2:** List of primers used in this study

PCR	Primer	Sequence (5′-3′)	Product size (bp)	PCR Cycles	Annealing temperature (°C)
**qRT-PCR**	Ecad-F	TACACTGCCCAGGAGCCAGA	103 bp	32	60
	Ecad-R	TGGCACCAGTGTCCGGATTA			
	Vimentin-F	GACCAGCTAACCAACGACAA	150 bp	32	60
	Vimentin-R	GTCAACATCCTGTCTGAAAGAT			
	SNAIL-F	GAGGCGGTGGCAGACTAG	178 bp	32	60
	SNAIL-R	GACACATCGGTCAGACCAG			
	PCDH17-F	GGGCAATCTGGACTATGAGG	108 bp	32	60
	PCDH17-R	ATGAGCTTGACCGTGACTTT			
	GAPDH-F	GGAGTCAACGGATTTGGT	206 bp	23	60
	GAPDH-R	GTGATGGGATTTCCATTGAT			
	c-Myc-F	GGAGGCTATTCTGCCCATTT	177 bp	32	60
	c-Myc-R	GTCGAGGTCATAGTTCCTGTTGG			
	cyclinD1-F	CTAGCAAGCTGCCGAACC	90 bp	32	60
	cyclinD1-R	TCCGAGCACAGGATGACC			
	β-catenin-F	TGTATGAGTGGGAACAGGGATT	190 bp	32	60
	β-catenin-R	GCCAAACGCTGGACATTAGT			
**BGS**	PCDH17-F	TGAGTAGAATAAGGAGAGATTAT	490 bp	40	60
	PCDH17-R	ACAACTAACACTTAACATTATAAC			

### DNA bisulfite treatment and methylation-specific PCR (MSP)

Bisulfite modifications of DNA and MSPs were performed as previously described [32, 33]. Bisulfite-treated DNA was amplified by MSP with primers PCDH17-m1 (5′-GATTATCGGGTGTCGTAGTTC-3′) and PCDH17-m2 (5′-CCCTAACGCAACGTACGCG-3′) for methylated samples, or PCDH17-u1 (5′-AGATTATTGGGTGTTG TAGTTT-3′)and PCDH17-u2 (5′-AACCCTAACACAA CATACACA-3′) for unmethylated samples. MSPs were conducted for 40 amplification cycles using AmpliTaq-Gold DNA Polymerase (Applied Biosystems), with annealing temperatures at 60 and 58°C for methylated and unmethylated samples, respectively. Methylated and unmethylated human DNA samples were used as positive and negative controls, respectively. Amplicons were analyzed following electrophoresis on 2% (w/v) agarose gels, which included 100 bp DNA markers (MBI Fermentas, Vilnius, Lithuania).

For BGS (bisulfite genomic sequencing), bisulfite-treated DNA was amplified using BGS primers (Table 2). The PCR products were purified and cloned into pCR4-Topo vector (Invitrogen, Carlsbad, CA), with 8 to 12 colonies randomly chosen and sequenced.

### Colony formation assays

MB231 and MCF7 cells were seeded in 6-well plates and transfected with an empty control vector, or a plasmid containing the full-length cDNA of *PCDH17*, using Lipofectamine™ 2000 (Invitrogen) according to the manufacturer's instructions. At 48 h post-transfection, cells were collected, re-plated, and selected for 2 weeks in the presence of 1.2 or 0.2 mg/mL G418 for MB231 and MCF7 cells, respectively. Survived colonies were counted following staining with Giemsa's solution.

### Wound healing assays and transwell assays

Following stable transfection, the cells were cultured in 6-well plates until they were confluent. Cultures were wounded using sterile pipette tips, washed twice with PBS, and re-suspended in fresh culture medium. MB231 and MCF7 cultures were incubated at 37°C/5% CO_2_ for 30 and 48 h, respectively. Cultures were then assessed at low power using a phase-contrast microscope (Nikon, Japan). Experiments were conducted in triplicate.

Cell migration was also assessed using transwell chambers (8-μm pore size, Corning, NY). MB231 cells stably expressing empty vectors or PCDH17 were washed twice in serum-free medium and plated onto transwell filter inserts in 24-well plates. The lower chambers contained 800 μL medium with 10% FBS. The cells were incubated at 37°C/5% CO2 for 24 h and fixed in 4% paraformaldehyde for 30 min, then stained with 100 ng/mL 4′,6′-diamidino-2-phenylindole hydrochloride (DAPI) for 15 min. Non-migratory cells in the upper chamber were removed with a cotton swab. Cells were photographed and counted under a microscope in five random fields.

### Immunofluorescence staining

Cells were cultured on glass coverslips and transfected with *PCDH17*. 48 h after transfection, cells were fixed for 10 min in 4 % paraformaldehyde in PBS (pH 7.4), then permeabilized for 4 min in 0.1% Triton X-100 in PBS. Cells were blocked for 1h with 1% bovine serum albumin in PBS and then incubated with primary antibodies against PCDH17 (Sigma-Aldrich, HPA026817), E-cadherin (Abcam, Cambridge, MA), and Vimentin (Abcam, Cambridge, MA) overnight at 4°C. Nuclei were counter stained with DAPI. Images were captured using a fluorescence microscope (Leica DM IRB).

### Analysis of cell cycle

To assess cell cycle status, MB231 and MCF7 cells were cultured in 6-well plates (2 × 10^5^ cells/well) and incubated at 37°C/5% CO_2_. Cultures were transiently transfected with 4 μg of pcDNA3.1(+)-Flag-PCDH17 plasmid or empty control vectors using Lipofectamine™ 2000 (Invitrogen, Carlsbad, CA, USA) according to the manufacturer's recommendations. After 48 h, cells were digested using 0.1% trypsin and centrifuged (900 rpm, 5 min). Cells were washed twice with PBS, fixed with ice-cold 70% ethanol for 1 h, and treated with 100 mL of 50 mg/L propidium iodide (PI) at 4°C for 30 min in the dark. Annexin V-FITC/PI staining was applied for apoptosis analysis. Data were analyzed using CellQuest™ Pro (BD Biosciences, San Jose, CA).

### Western blotting analysis

At 48 h post-transfection, cells were harvested and lysed with M-PER Mammalian Protein Extraction Reagent (Pierce, Cramlington, UK) containing a protease inhibitor cocktail (Sigma-Aldrich). Proteins in cell lysates (50 μg of total protein) were separated by sodium dodecyl sulfate polyacrylamide gel electrophoresis and then transferred to polyvinylidene difluoride membranes (Bio-Rad, Hercules, CA, USA). We used primary antibodies against PCDH17 (Sigma-Aldrich), active β-catenin, total β-catenin, c-Myc, cyclin D1, cyclin B1, p27, p21 (Cell Signaling Technology, USA) and GAPDH (Southern Biotech, Birmingham, AL, USA). Protein bands were visualized using an enhanced chemiluminescence kit (Amersham Pharmacia Biotech, Piscataway, NJ, USA).

### *In vivo* tumorigenicity

4-week-old female BALB/c nude mice (6 mice/group) were injected subcutaneously with vector- or *PCDH17*- stably transfected MB231 cells (5×10^6^). Tumor volume (mm3) was calculated using the following equation: volume = 0.5 × length × width^2. Before the length of the largest tumor reached 1cm, all mice were sacrificed and the tumors were removed and weighed. For immunohistochemical analyses, paraffin-embedded sections were prepared from the xenografts. Proliferation score was calculated by counting the number of Ki67-positive cells in 100 tumor cells in 10 randomly selected fields on one slide under x 400 magnification. All procedures were approved by the Animal Ethics Committee of the Experimental Animal Center of the Chongqing Medical University, Chongqing, China.

### Statistical analysis

Statistical analyses were performed with SPSS version 16 (SPSS Inc., Chicago, IL, USA). Student's *t*-test, χ^2^ tests, and Fisher's exact test were used to compare methylation status and clinicopathological parameters. For all tests, a *p*-value less than 0.05 was considered statistically significant.
